# Virtual action and real action have different impacts on comprehension of concrete verbs

**DOI:** 10.3389/fpsyg.2015.00176

**Published:** 2015-02-24

**Authors:** Claudia Repetto, Pietro Cipresso, Giuseppe Riva

**Affiliations:** ^1^Department of Psychology, Catholic University of Sacred HeartMilan, Italy; ^2^Applied Technology for NeuroPsychology Lab, Istituto Auxologico ItalianoMilan, Italy

**Keywords:** embodied language, virtual reality, comprehension, action verbs, abstract verbs

## Abstract

In the last decade, many results have been reported supporting the hypothesis that language has an embodied nature. According to this theory, the sensorimotor system is involved in linguistic processes such as semantic comprehension. One of the cognitive processes emerging from the interplay between action and language is motor simulation. The aim of the present study is to deepen the knowledge about the simulation of action verbs during comprehension in a virtual reality setting. We compared two experimental conditions with different motor tasks: one in which the participants ran in a virtual world by moving the joypad knob with their left hand (virtual action performed with their feet plus real action performed with the hand) and one in which they only watched a video of runners and executed an attentional task by moving the joypad knob with their left hand (no virtual action plus real action performed with the hand). In both conditions, participants had to perform a concomitant go/no-go semantic task, in which they were asked to press a button (with their right hand) when presented with a sentence containing a concrete verb, and to refrain from providing a response when the verb was abstract. Action verbs described actions performed with hand, foot, or mouth. We recorded electromyography (EMG) latencies to measure reaction times of the linguistic task. We wanted to test if the simulation occurs, whether it is triggered by the virtual or the real action, and which effect it produces (facilitation or interference). Results underlined that those who virtually ran in the environment were faster in understanding foot-action verbs; no simulation effect was found for the real action. The present findings are discussed in the light of the embodied language framework, and a hypothesis is provided that integrates our results with those in literature.

## INTRODUCTION

Imagine being in a cinema, looking at an action movie. The protagonist keeps running through the streets and jumping from one car roof to another, trying to escape from his enemies, who are in pursuit to kill him. What happens in our brain in this moment? Thanks to the activation of the mirror neurons system (Rizzolatti and Craighero, 2004), a phenomenon occurs, that is often referred to as motor resonance: when I see someone doing something, his/her action produces a “resonance effect” in my brain, as if I were doing that action myself. Motor resonance has been widely described in many experimental studies about action observation (Greenwald, 1970; Jeannerod, 1994; James and Maouene, 2009). However, there are empirical data suggesting that motor resonance is triggered also by action-related linguistic stimuli (Gentilucci and Gangitano, 1998; Gentilucci et al., 2000; Gentilucci, 2003; Glover et al., 2004; Tucker and Ellis, 2004; Zwaan and Taylor, 2006). This view is in agreement with the theoretical framework called Embodied Cognition (Barsalou, 2008), which puts forward that the process of understanding a sentence brings about a language-induced mental simulation of the actions described in the sentence.

In the last decade, many data have been reported that support the hypothesis of a simulation-based language comprehension. However, the direction of the effect of the simulation process is still unclear: does simulation help or interfere with language processing? The answer to this question is not yet obvious. In literature, there are studies reporting contradictory results. In some cases, the simulation process is deemed to produce faster RTs, thereby having a facilitation effect. Findings of this kind are common: Myung et al. (2006) found a facilitation in lexical decision about functionally similar objects (piano-typewriter); Rueschemeyer et al. (2010) reported faster RTs when the action required to give the response matched that described by the linguistic stimulus (toward vs. away from the body); Glenberg et al. (2008) reached analogous conclusions also with abstract content sentences, describing transfer of information; Zwaan and Taylor (2006) and Taylor and Zwaan (2008) arrived at similar findings by using action stimuli related to rotation (clockwise vs. counter clockwise).

Conversely, the reverse situation is also described, characterized by an interference effect due to the match between the effector used to provide the answer and that involved in the action word or sentence processed. For example, Buccino et al. (2005), using a go/no-go response during a semantic decision task, found that the match between the effector employed to give the response (hand vs. foot) and that ideally used to perform the action described by the verb (hand-related vs. foot-related verbs) resulted in slower responses than in case of mismatch. Similarly, an interference occurred in the studies by Sato et al. (2008), who ran three behavioral experiments with a go/no-go task. In the first two, a semantic comprehension task was required, but with early versus late delivery of the go signal; in the third one, the task required was a lexical decision. Authors found an interference effect only for the semantic task (Experiment 1), and only when the signal was delivered while the semantic comprehension was occurring (early delivery).

Thus, the aim of the present work is to investigate the simulation process using a traditional paradigm, but in a novel experimental setting: virtual reality. In particular, we want to test if simulation could be achieved also performing a virtual action (an action performed within a virtual environment with a body part which is actually steel).

Virtual reality (VR) is a combination of technological devices that allow users to create, explore and interact with 3D environments. Typically, an individual entering a virtual environment feels a part of this world and has the opportunity to interact with it almost as he/she would do in the real world: a user can visually explore the scene just by turning his head, and manipulate other user-friendly controls to move through the environment, approach objects, select them, meet other people (presented as avatars).

The connection of the virtual experience to the real world relies mostly on three features: sight, hearing, and interaction. The visual input in most cases is provided by means of a computer monitor or a head-mounted display (HMD). The HMD is a visualization helmet that conveys the computer-generated images to both eyes giving the illusion of a third dimension in the surrounding space. Aural devices may be head-based, like headphones, or stand-alone, like speakers.

Traditionally the most common application of VR in mental health is related to the treatment of anxiety disorders (Repetto and Riva, 2011), but in recent years, the use of this tool in the field of neuroscience (Bohil et al., 2011) has received growing attention. In particular, VR is a great opportunity for researchers interested in studying cognitive processes from an embodied point of view: if representations in the cognitive system are multimodal (Barsalou, 2008), then to investigate their properties, one should recreate the multimodal experience that can trigger the process. Furthermore, with advancements in technology, the interface between subject and VR system is more and more designed as a non-mediated process, in which the body itself will be the navigation tool (without the need of control devices – please note that in this research this feature has not been implemented). For these reasons, VR is an ideal medium for investigating several cognitive domains (Riva, 1998). It should be noticed that little is known so far about the brain correlates of the virtual action (Wagner et al., 2014), and this gap should be filled in order to build a global theoretical framework. Nonetheless, we argue that researchers who use actions for understanding the interplay between language and the motor systems would find implementation of VR to be an advantageous medium (Repetto, 2014). VR gives users the opportunity to see themselves moving in the environment while comfortably seated in a chair. Thanks to different input devices, participants can virtually perform any action, even those typically not performable in an experimental setting (jump a rope, kick a ball, shoot a gun, etc). Thus, within a virtual environment, experimenters can investigate the effect on language processing of performing different actions. The fact that users are not really moving their bodies in real space, but still have the sensation of being “in action,” places VR in a intermediate position between real physical action and action observation (such as in a video). It has been demonstrated that cortical excitability is modified by the observation of movements performed by others (Strafella and Paus, 2000), but this modulation is greater if the orientation of the movement is compatible with the point of view of the observer (Maeda et al., 2002). The advantage of VR is that the movement of the individual is egocentric, exactly as he/she would act in the real world. As Cameirao et al. (2010) has argued, the first person perspective could strongly engage the mirror neurons system because this is the perspective the system is exposed to most frequently.

The present study is a pioneer protocol that challenges the capabilities of VR in the domain of language. The paradigm used is replicated from the study by Buccino et al. (2005); the innovation is the use of a virtual world that allows the user to have the impression of performing an action with a body part, which is actually completely steel. Participants were placed in a virtual environment in which they had to perform a semantic task (concreteness judgment): in one condition they ran in the virtual park by moving the joypad knob with their left hand (virtual action performed with their feet plus real action performed with the hand) and in the other condition they only watched a video of runners and executed an attentional task by moving the joypad knob with their left hand (no virtual action plus real action performed with the hand).

Thus, the specific purpose of this study is to test which action (the virtual one or the real one) triggers simulation. The second related goal is to determine the direction of the effect (facilitation vs. interference): in particular, we want to know if the virtual action is effective in inducing motor simulation. This outcome, in fact, would be particularly interesting since it would open new avenues in the study of the relationships between action and language.

The following predictions can be outlined:

– the actual motion yields a simulation effect, and, based on the previous literature, supposedly it will be an interference: if so, all the participants (since they use their hands to give the response to the linguistic task) should be slower in providing the response to the hand-action verbs;
– the virtual motion as well (thanks to its first-person perspective) produces a simulation effect: if so, participants who virtually ran in the environment, should show a performance profile different from those who only watched a video of runners, selectively for foot-action verbs.

## MATERIALS AND METHODS

### PARTICIPANTS

Twenty four volunteers, (10 males and 14 females; age: range 23–45 years; mean: 35.71; years of education: range 13–19; mean: 15.88) were recruited for the experiment via public advertisement, and the subsequent snowball effect. Participants were all native Italian speakers, right-handed (Briggs and Nebes, 1975), with normal or corrected-to-normal vision, and no history of neurological or psychiatric diseases. Someone was still student, others had a work. None of them was aware of the specific purpose of the study. All of them signed an informed consent in order to join the experiment. The experimental procedure, and the specific consent form describing it, had been previously approved by the University Ethic Committee.

### STIMULI

Twenty sentences were constructed for each type of verb: hand-action verb, foot-action verb, mouth-action verb, and abstract verb. We used the same set of sentences used by Buccino et al. (2005), plus a number of new ones related to mouth-action verbs. The choice to introduce mouth-action verbs is motivated by the need to have a set of action verbs whose effector was not involved either in the real or in the virtual action. Sentences containing hand-action verbs, foot-action verbs or mouth-action verbs were considered concrete-content sentences, expressing a concrete action performed with different effectors (respectively, hand, foot, and mouth). On the other hand, sentences containing abstract verbs were considered abstract-content sentences, typically expressing intellectual or symbolic activities. Each sentence was repeated from two up to six times; on the whole, forty sentences for each type of verb were presented, thus the experiment consisted of 160 trials. **Table 1** reports the list of sentences, specifying for each one the number of repetitions.

**Table 1:** The complete list of items (and their English translation).

Hand action verb	Foot action-verb	Mouth action-verb	Abstract verbs
Cuciva la gonna (2) (He) Sewed the skirt	Calciava la palla (4) (He) Kicked the ball	Baciava la guancia (6) (He) Kissed the cheek	Amava la moglie (2) (He) Loved his wife
Gairava la chiave (2) (He) Turned the key	Calciava la porta (4) (He) Kicked the door	Baciava la mamma (4) (He) Kissed the mom	Amava la patria (2) (He) Loved his country
Lavava i vetri (4) (He) Washed the windows	Calciava la sedia (2) (He) Kicked the chair	Leccava il francobollo (6) (He) Licked the stamp	Gradiva la mela (4) (He) Loved the apple
Prendeva la tazza (2) (He) Took the cup	Correva nel parco (4) (He) Run in the park	Leccava il gelato (4) (He) Licked the ice-cream	Odiava il mare (4) (He) Hated the sea
Scriveva il tema (2) (He) Wrote the essay	Correva sul prato (4) (He) Run over the grass	Mordeva il pollo (6) (He) Bit the chicken	Pativa il caldo (2) (He) Suffered from the heat
Sfilava il filo (2) (He) Paraded the thread	Marciava sul posto (4) (He) Marched on the place	Mordeva la pagnotta (4) (He) Bit the bread	Perdeval la guerra (2) (He) Lost the war
Sfiogliava il libro (2) (He) Turned over the pages of the book	Pestava l’erba (4) (He) Trod on the grass	Succhiava il latte (4) (He) Sucked the milk	Perdeva la pazienza (2) (He) Lost his patience
Spalmava la crema (2) (He) Spread the cream	Pestava la corda (2) (He) Trod on the rope	Succhiava il pollice (6) (He) Sucked the thumb	Sapeva la poesia (4) (He) Learned the poem
Spezzava il pane (4) (He) Broke the bread	Pestava le foglie (4) (He) Trod on the leaves		Scordava il nome (2) (He) Forgot the name
Stringeva la mano (2) (He) Shook the hand	saltava il fosso (2) (He) jumped the ditch		scordava la data (2) (He) forgot the date
Suonava il piano (2) (He) Played the piano	Saltava il muro (4) (He) Jumped the wall		serbava l’odio (4) (He) kept the hate
Svitava il tappo (4) (He) Unscrewed the stopper	Saltava la corda (2) (He) Jumped the rope		Soffriva il freddo (4) (He) Suffered from the cold
Tagliava la carne (2) (He) Cut the meat			Temeva il buio (2) (He) Feared the dark
Tagliava la stoffa (2) (He) Cut the cloth			Temeva la pena (2) (He) Feared the penalty
Timbrava la busta (2) (He) Stamped the envelope			Vinceva la gara (2) (He) Won the competition
Stappava la bottiglia (2) (He) Uncorked the bottle			
Firmava il contratto (2) (He) Signed the contract			

*In parenthesis the number of repetitions for each item, for a total of 160 items presented in a single block*.

The sentence’s syntactic structure was the following: verb + complement (article or preposition plus the appropriate object, for a total of three words). The verbs were all formed by three-syllables and were conjugated at the third person of the simple past tense, which requires the suffix –va to be added to the verb stem. The frequency of use of the verbs in the four types of sentences was kept similar, based on the available data about the frequency of use norms for the Italian language (De Mauro et al., 1993).

#### Virtual environment

The virtual environment was launched through the freeware software NeuroVr2 (http://www.neurovr2.org; Riva et al., 2009). It was designed to be a park on a sunny day. When entering it, the participant started on a paved track, and the first-person point of view was set up as for an adult standing, ready to explore the park. Outside the track, the ground was completely covered by green grass, and enriched with trees and shrubs. In addition to natural items, there were many artifacts, which one would typically encounter in a park: benches, streetlamps and bins. A picnic area and a playground were displayed. No human being was present in the scene. The paved track circled around the two above-mentioned areas, and then led to a hill where the edge of the environment was set up. From the top of the hill, one side looked down on the park, and the other side displayed fog that indicated the end of the area where exploration was allowed.

All the objects, both natural and artifacts, were true solid entities that could not be passed through; just as in the real world, if the user accidentally or purposely banged into one of them, his or her walk was stopped until he/she changed direction.

**FIGURE 1:**
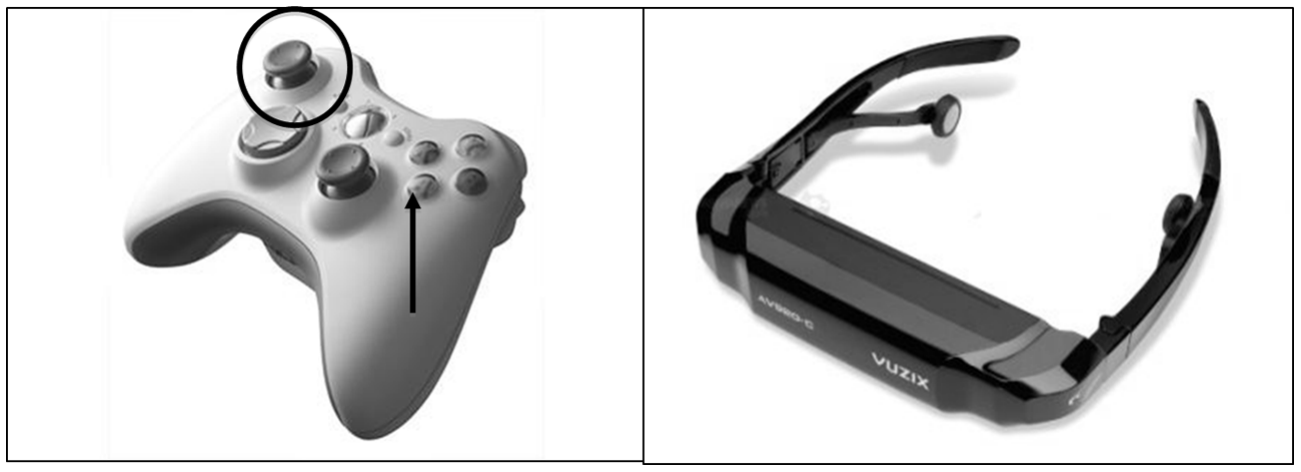
**Tools used to explore VR.** On the left: the Xbox 360 joypad; the circle indicates the knob used to walk in the virtual environment, and the dart the key pressed to give the response. On the right: the Vuzix AV920 head-mounted display (HMD).

The interaction with the environment (when required, depending on the experimental condition – see below for a detailed description) was regulated by manipulating the left knob of the joypad (Xbox 360; see **Figure 1**, left side): moving it in the forward/backward or left/right directions provided a coherent movement in the virtual scene. The key A was pressed to give the appropriate response when needed (see the next section for the procedure’s description). The HMD (Vuzix AV920: see **Figure 1**, right side), together with the connected headphones, allowed an immersive experience.

### PROCEDURE

During the experimental protocol, an experienced researcher welcomed the participants into a quiet room. After reading and signing the informed consent, they began the experimental task. The VR equipment included the PC, on which the virtual scene was displayed, and the interactive tools (joypad and HMD): it was all arranged in front of the participant at a distance of approximately 50 cm.

Once the electrophysiological tools were arranged, the participants wore the HMD and held the controller, while the researcher launched the practice session to familiarize the participant with the environment and the commands needed to interact with it. Next, the experimental session started. The main task was a semantic judgment of sentences presented auditorily. Participants were instructed to perform a go/no-go task, in which they had to press a key on the joypad when the sentence heard was a concrete-content one, and refrain from pressing when the sentence heard was an abstract-content one. The go signal was a flash presented visually as a transient change of the light in the environment; it occurred the first time 10 s after entering the environment and then every 5 s, always in coincidence with the end of the second syllable of the verb (e.g., corre’va sul prato) that is, approximately 500–700 ms after the beginning of the sentence, depending on the verb’s length (the position of the go signal was synced working on the voice spectra, by moving the sentence until the target position was reached). The response key was that identified by the dart in **Figure 1**, and it was pressed with the right thumb.

**FIGURE 2:**
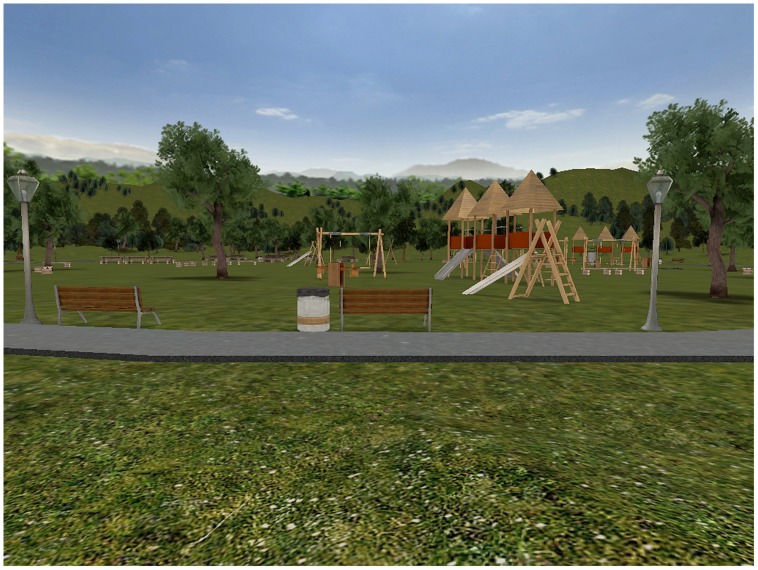
**A screenshot of the virtual park displayed in the Run condition**.

In addition to the main task, the participant had to follow different instructions according to their designated experimental condition. Participants were randomly assigned to one of the two experimental conditions, which differed in terms of degrees of action: run and video conditions. In the run condition, the participants performed the main task (semantic comprehension) while exploring the park as if they were walking or running through it (**Figure 2**). The specific instructions stressed that they had to keep walking in any direction without stopping until the sentences ended. The walk-like action inside the park was obtained by moving the joypad knob on the left (see the circle in **Figure 1**) with their left hand: in this way the optic flow changed coherently giving the impression of walking trough the environment. This experimental condition required people to stand in front of the computer in order to assume a body position coherent with the virtual walk.

**FIGURE 3:**
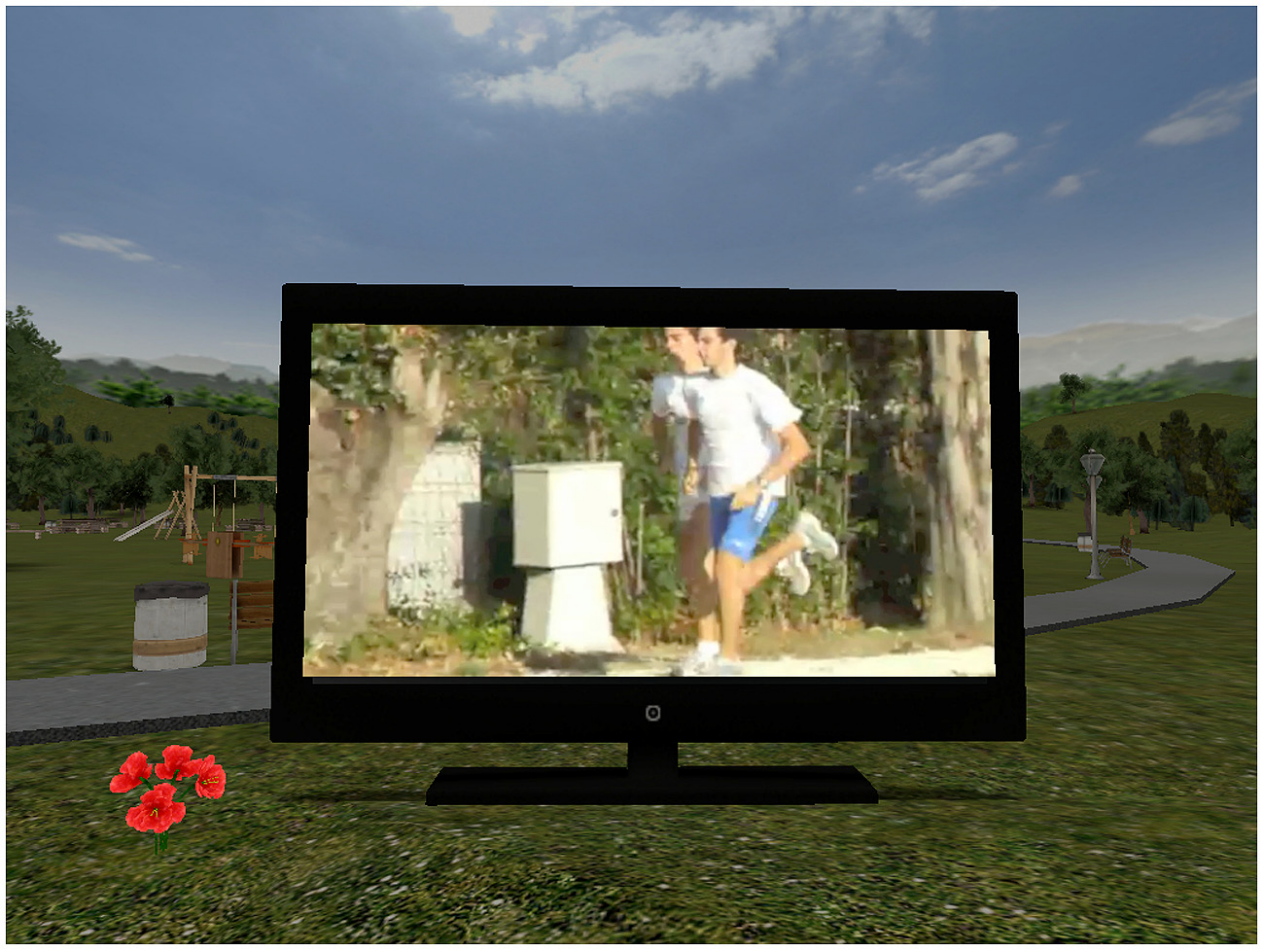
**A screenshot of the virtual park displayed in the Video condition**.

In the video condition, the participants sat in front of the computer and began the virtual experience as if they were seated on a bench. In front of them, in the virtual environment, they could see a television displaying a video of runners (**Figure 3**). The participants were instructed to look at the video carefully and to move the left knob when the direction of the motion in the video changed. This assignment was done in order to pursue two goals: on one side, to make this condition comparable to the previous one in terms of attentional load, and to assign a task to the left hand; on the other side, to be sure that the participants continuously watched the video content. This task was performed in concomitance with the main comprehension task. In sum, all the participants had to perform the main task (semantic comprehension) with the right hand (by pressing the key when needed) while performing a second, visuospatial task, with the left hand (by moving the knob).

The experimental session took about 13 min.

### DATA RECORDING AND ANALYSIS

Electromyography (EMG) latencies were used as a measure of the behavioral task; this choice was made, on one hand, in order to collect very precise and reliable RT data; on the other hand, it was necessary in order to sync the different sources of stimuli (VR and audiotape) with participants’ responses. The raw EMG is a collection of positive and negative electrical signals; generally, the root mean square (RMS) is considered for rectifying the raw signal and converting it to an amplitude envelope (Blumenthal et al., 2005). There are a number of measures that can be extracted from this signal that depend on the muscle corresponding to the electrode’s location. For this study, we considered the RMS of EMG signals acquired by two patches placed on the flexor pollicis brevis muscle, which is involved in the button pressing; one additional reference patch was placed on the arm for reference.

## RESULTS

The experimental design of the study was a mixed design, with one independent variable within subjects with three levels (Verb: hand – mouth – leg/foot), and one independent variable between subjects with two levels (Condition: Run – Video). **Table 2** reports descriptive data.

**Table 2:** Descriptive data.

Verb type	Condition	*N*	Mean	SD	SE	Minimum	Maximum
Mouth-action verbs	Video	12	320,08	102,439	29,572	163	447
	Run	12	262,58	110,863	32,003	110	439
	Total	24	291,33	108,441	22,135	110	447
Foot-action verbs	Video	12	355,67	112,919	32,597	131	493
	Run	12	244,42	86,006	24,828	146	429
	Total	24	300,04	113,422	23,152	131	493
Hand-action verbs	Video	12	314,33	88,383	25,514	198	471
	Run	12	316,33	106,333	30,696	126	500
	Total	24	315,33	95,627	19,520	126	500

The first analysis was performed in order to verify the effects of Verb toward the dependent variable (RTs) separately for the two experimental conditions. Repeated Measures ANOVA test highlighted no differences in either the Run or the Video condition for any type of verbs (Video condition, *F*_(2,22)_ = 0.743; *p* = 0.487; η^2^= 0.06; Run condition, *F*(2,22) = 1.568; *p* = 0.231; η^2^ = 0.12).

As a second step, we were interested in comparing the performances for different types of verbs between the two groups. To do that, we conducted a One Way ANOVA test, considering Group as a between subjects variable. RTs for foot action-verbs were significantly faster in the Run condition than in the Video condition [*F*_(1,22)_ = 7.371; *p* = 0.013; η^2^ = 0.25], whereas the hand and mouth action-verbs were processed similarly in the two groups [*F*_(1,22)_ = 0.003; *p* = 0.96; η^2^ = 0.00 and *F*_(1,22)_ = 1.741; *p* = 0.201; η^2^ = 0.07, respectively].

**FIGURE 4:**
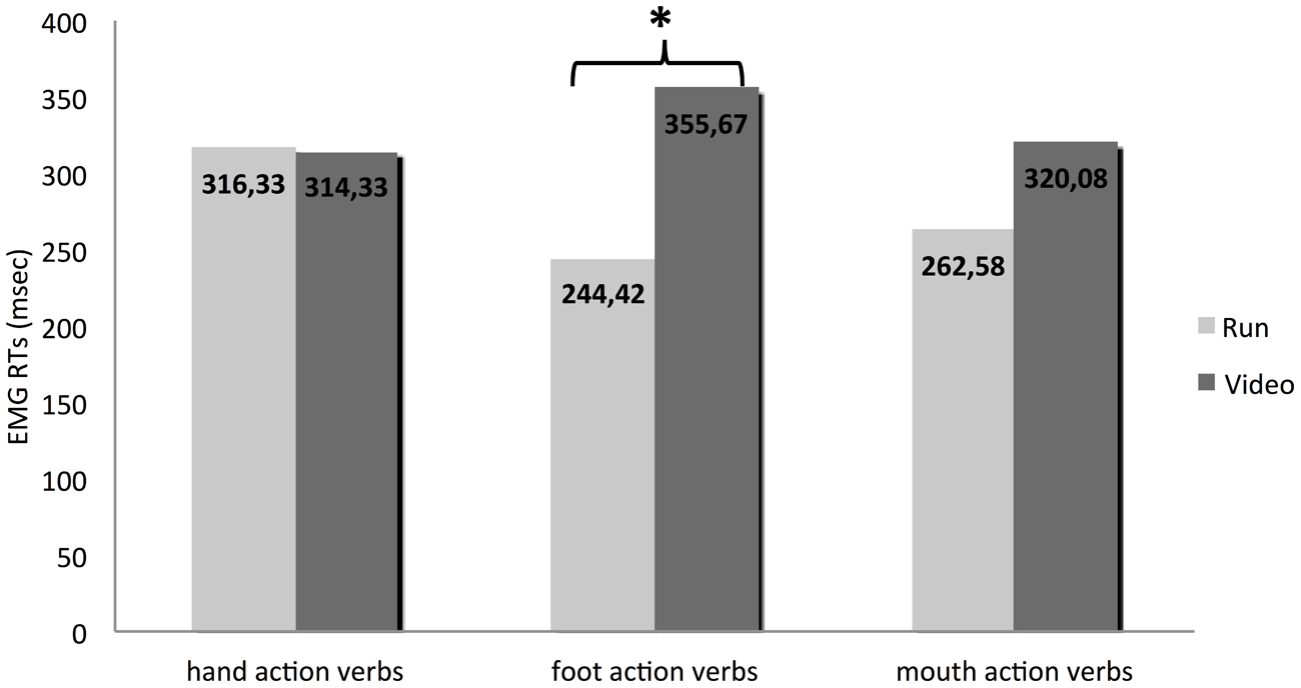
**The performances of the two groups for each type of verb.** * indicates a difference statistically significant.

**Figure 4** illustrates the performances of the two groups for each type of verb.

## DISCUSSION

The present experiment aimed to extend the knowledge of simulation in language comprehension, by using a traditional paradigm but with novel experimental tools, thanks to VR technology. For this purpose, we set up an experimental apparatus that included tools traditionally used in neuroscience EMG, and other borrowed from positive and general psychology research (virtual reality). Combining these different tools required a strong effort, mainly in the synchronization process, that allowed the measures and the stimulation to be provided simultaneously and recorded precisely. Results underlined that the match between the effector described by the verb and that engaged in the virtual action resulted in faster linguistic processing of the sentence, thus suggesting a facilitation effect; on the other hand, no interference effect arose from the match between the effector described by the verb and the that used to provide the response.

According to the present findings, we can at least partially answer the research question about the feasibility of VR in triggering simulation and about the direction of this effect (the virtual action seemed able to trigger simulation and influence language performance, whereas the real action was not; furthermore, the direction of the effect underlined by the present findings is a facilitation).

The critical point is the failure to recognize significant differences in processing hand/mouth/foot action-verbs within the group: an initial, but naïve, explanation would be to postulate the absence of a simulation process, thus considering that, in this paradigm, the motor system and the linguistic stimulus did not interact at all. This lack of effect could be better explained by taking into account the experiment structure: the number of repetitions of the same verb, which ranged from a minimum of 2 to a maximum of 10, considering that the same verb was used in different sentences, could have lead to a priming effect, thus facilitating the comprehension of the sentence and masking the possible concomitant simulation effect. The impact of the stimulus repetition proportion, in fact, has recently been pointed out by Britt et al. (2014), who found that a higher proportion of linguistic target repetition yielded more language-driven anticipatory eye movements. According to this latter explanatory hypothesis, the study design could account for the lack of within-groups differences: the high rate of similar linguistic structures could have leveled the response times for all the types of verbs, covering the potential effect due to the match/mismatch between the effector used to provide the answer and the effector described by the verb processed.

In support of this view is the second, perhaps more interesting, result: when comparing the different conditions, with and without virtual movement, a clear effect arises, indicating that participants who virtually walk/run in the environment processed foot-action verbs faster than those who only watched a video of runners. Hand and mouth action verbs were processed similarly in both groups. Thus, based on these findings, we can consider that our data support the hypothesis that the virtual action is able to induce a simulation process that impacts semantic comprehension, and the direction of this effect is compatible with a facilitation; however, previous findings by Buccino et al. (2005) were not replicated in our experiment, since the real movement, performed with the hand, did not interfere with the comprehension of hand action-verbs.

These results raise at least three theoretical questions: why and how can VR trigger motor simulation? Why does it appear as a facilitation effect and not as an interference as previously found in similar settings, but with real actions? Why, in this setting, does the real action have no effect at all?

The first issue should be addressed starting from the basic concepts of the embodied language position. According to it, the motor system is involved not only in action execution, but also in linguistic processing of action words: thus, language and motor system seem connected, and even influence each other bidirectionally. In particular, the primary motor cortex (M1) seems to play a role in language comprehension: a temporary reduction of the cortical excitability of the portion of M1 that controls the hand results in slower comprehension of hand action-verbs (Repetto et al., 2013). This is important if we consider that M1, during action observation, is activated differently depending on the observer’s point of view, as reported by Maeda et al. (2002). In their work, participants viewed video of hand movements presented from two points of view: one compatible with the observer position, and one incompatible with it. Cortical excitability in the two conditions was measured by means of TMS stimulation and registration of MEP of hand muscles. Data underlined how, as already reported in other studies (Fadiga et al., 1995; Strafella and Paus, 2000), the action observation induces changes in cortical excitability; but, more interestingly, MEP facilitation was higher when the observed action matched the observer’s point of view. It means that the observation-induced motor cortical modulation is modified by the action’s orientation.

In our experimental setting, the virtual action of run was observed by the participants as if they were the actors, from the first person point of view: even if no body parts were visible in the environment, the subjective feeling of motion was guaranteed by the coherent change of the visual field in the virtual world. The importance of the virtual experience in activating cortical regions usually deputed to motor planning and motor intention has been recently described by Wagner et al. (2014), who found increased activity in premotor and parietal cortex when participants virtually walked in a valley, compared to other kinds of visual feedback (unrelated feedback and the person’s image perceived as in a mirror).

So we can suppose that VR, taking advantage of the first person point of view, is able to modulate M1 excitability more than the mere observation of someone else’s action. Even if imaging studies are needed to confirm this hypothesis, current knowledge suggests that VR can trigger simulation, since it possibly recruits the same cortical regions involved in language processing as well as in action execution.

Given that the contribution of VR in promoting motor simulation could be accounted for by its ability to elicit the first person point of view, the direction of the effect still needs to be discussed. As previously reported, literature showed that different experimental paradigms (Boulenger et al., 2006; Dalla Volta et al., 2009) sometimes led to contradictory results. In general, when the focus of the research was the direction of movement, such as in the typical Action Sentence Compatibility Effect (ACE) designs (Glenberg and Kaschak, 2002), the match between the action and the sentence or word meaning resulted in a facilitation; but conversely, when the focus was the effector, the match between the effector performing the action and that described by the verb resulted in either facilitation (Pulvermuller et al., 2001; Hauk et al., 2004; Tettamanti et al., 2005; Scorolli and Borghi, 2007) or interference (Buccino et al., 2005; Bergen et al., 2010; Mirabella et al., 2012). Our findings fit in the second class of studies: the paradigm was focused on the match/mismatch of the effector and the simulation seemed to produce facilitation.

Nevertheless, it is possible to integrate the present finding with the literature data and reconcile the contrasting results. One way is that proposed by Chersi et al. (2010): authors modeled a neural mechanism able of explaining the interaction between action and language in both terms of facilitation and inhibition. According to this model, based on the neural dynamics of the parietal and premotor cortices (Fogassi et al., 2005), the interaction effects arise as facilitation or interference depending on the timing between the stimulus presentation and the action required.

An alternative explanation is arguing that the interplay between the meaning and the motor programs changes crucially depending on the movement features. When an action word must be understood, motor areas play a functional role (Willems et al., 2011; Repetto et al., 2013) supporting the linguistic process: if a concomitant action must be performed, then the properties of that action could predict different outcomes. If I am processing a hand word/sentence and I have to move my hand at the same time, the hand portion of my motor areas are involved in two processes, one linguistic and one truly motor. In this case, we think that, as originally suggested by Buccino et al. (2005) the motor programs needed to execute the action and deliver it to the muscles could compete with the simulation of the action described by the linguistic stimulus, resulting in an interference effect. The opposite could happen if the motor programs needed to execute the action are compatible with those described by the verb, as typically occurs in the case of ACE paradigms: in these studies the same portion of the motor cortex is supposed to support both – linguistic and motor – processes, but the former possibly acts as a prime to facilitate the latter. In the VR paradigm, the motor cortex is available and potentially preactivated by the virtual motion, but with no commitment to produce a real movement, there is no requirement to deliver neural signals to activate the muscles. Maybe this condition of alertness, without execution, again acts as a prime, resulting in faster responses.

Finally, the lack of interference effect during real action (hand movement) deserves some attention. As stated before, the paradigm used in this research essentially replicated that by Buccino et al. (2005), thus the prediction was to find the same interference effect when there was a match between the effector used to provide the response and the effector described by the verb. Surprisingly, it did not occur; however, a careful consideration of the experimental setting and design could help us account for this anomaly. In our study, differently from Buccino’s, in addition to the motor response and the action verb, there was always a third motor cue: the virtual walk/run in the condition run, and the observation of others’ run in the video condition. It should be noticed that this third motor information was highly relevant for the task in both conditions (i.e., participants had to pay attention to the virtual walk in order to move continuously in the environment or to the runners in order to give the correct response when they changed direction). The literature often has pointed out how different task conditions related to the linguistic task led to different results in performance (Mirabella et al., 2012; Diefenbach et al., 2013); we can argue that the same happens when different task conditions and requirements are associated with the motor task.

## CONCLUSION

The present experiment was designed with an innovative experimental apparatus in order to deepen the knowledge of the simulation process, taking advantage of VR technology. The combination of a fully controlled psycholinguistic paradigm with the VR apparatus has been challenging, and entailed some limitations along with new opportunities and perspectives. One of the limitations is that we did not compare different kinds of virtual movements, nor the virtual movement with the correspondent real one. This enriched paradigm would have allowed us to formulate predictions that are more precise and to draw better conclusions, but at the same time would have required a more sophisticated technology, affecting the participants’ comfort. The second limitation is the small pool of verbs, repeated over time to build the complete set of items: unfortunately, this problem cannot be easily resolved, since there are not that many verbs describing actions performed uniquely with a specific body part.

Looking at opportunities, a completely new finding arising from this study is the impact of VR in cognitive processing: a virtual motion can elicit motor simulation and influence linguistic processing. This outcome is important for at least two reasons. First, it encourages further researches in the same direction, oriented to continue the investigation of the link between action and action-related language, but with a new tool – virtual reality. Second, it opens new paths toward the rationale for using VR in rehabilitation contexts: if the virtual motion acts on the brain similarly to the real one, supporting comprehension, but without the side effects of interference in case of competing movements, this represents an opportunity for the rehabilitation of language, especially for those patients who suffer from motor disabilities.

Surely, several further researches are needed to better understand the cognitive and motor representations triggered by a virtual experience, starting from the investigation of the neural correlates of the virtual actions; furthermore, it would be interesting to compare different virtual actions (performed with different body parts) with their real counterparts, in particular in relation to their capabilities to trigger simulation and influence language processing.

## Conflict of Interest Statement

The authors declare that the research was conducted in the absence of any commercial or financial relationships that could be construed as a potential conflict of interest.
